# Circ_0006174 promotes the malignancy of colorectal cancer cell via the miR-1205/CCBE1/Wnt pathway

**DOI:** 10.3892/mmr.2022.12767

**Published:** 2022-06-07

**Authors:** Xun Zhao, Dejun Cui, Fang Yan, Liuchan Yang, Manman Zhang, Bo Huang

**Affiliations:** Department of Gastroenterology, Guizhou Provincial People's Hospital, Medical College of Guizhou University, Guiyang, Guizhou 550002, P.R. China

**Keywords:** colorectal cancer, circ_0006174, calcium-binding epidermal growth factor domain-containing protein 1, miR-1205

## Abstract

Circular RNAs (circRNAs) are novel RNA transcripts that participate in cancer development. Nonetheless, in colorectal cancer (CRC), the information ~circRNA expression and function is largely unknown. The present study aimed to investigate the expression, function and underlying mechanism of circ_0006174 in CRC. Reverse transcription-quantitative PCR analysis was performed to detect circ_0006174, miR-1205 and calcium-binding epidermal growth factor domain-containing protein 1 (CCBE1) expression levels in CRC tissues and cell lines. Circ_0006174 knockdown CRC cell models were established. CCK-8, TUNEL and Transwell methods were utilized to explore the function of circ_0006174 on the malignant phenotype of CRC cells. Moreover, a xenograft nude mouse model was constructed to verify the effects of circ_0006174 on lung metastasis *in vivo*. Dual-luciferase reporter gene assay was adopted to prove the association between circ_0006174 and miR-1205, miR-1205 and CCBE1. Gene set enrichment analysis was performed using the LinkedOmics database. Western blotting was performed to evaluate the expression of CCBE1, Ki67 and Wnt pathway-related proteins (c-Myc and cyclin D1) in CRC cell lines. Circ_0006174 showed a notable upregulation in CRC tissues and cell lines and its overexpression was linked to larger tumor diameter and advanced T stage of CRC patients. Circ_0006174 knockdown significantly suppressed cell growth and metastatic potential and promoted cell apoptosis *in vitro*. Circ_0006174 knockdown accelerated the lung metastasis *in vivo*. Mechanistically, circ_0006174 could decoy miR-1205 to up-modulate CCBE1 expression and Wnt pathway-related proteins (c-Myc and cyclin D1). Circ_0006174 is an oncogenic circRNA, which participates in the promotion of CRC progression by regulating the miR-1205/CCBE1/Wnt pathway.

## Introduction

Colorectal cancer (CRC) is a common tumor. Annually, there are more than 600,000 CRC deaths worldwide and, at the time of diagnosis, ~25% of CRC patients have already suffered from distant metastases (1). With the advancement of CRC diagnosis and therapy, the 5-year survival rate of stage I and stage II patients has been improved to >82% (2). Nonetheless, the prognosis of CRC patients with advanced disease is still unfavorable and the 5-year survival rate is ≤10% (3). Hence, it is imperative to clarify the molecular mechanism of CRC carcinogenesis and progression to seek innovative targets for diagnosis and therapy.

Circular RNAs (circRNAs) are non-coding (nc) RNA transcripts that have a stable circular structure and are expressed in diverse cells. They can work as competing endogenous RNAs (ceRNA) to down-modulate microRNA (miRNAs/miRs) expression and take part in the tumorigenesis and development of diverse tumors (4). Reportedly, circRNAs are implicated in the tumorigenesis and metastasis of CRC and can be used as potential targets for CRC diagnosis and therapy, such as circ_0007031 (5), circRAE1 (6), circ_0007142 (7). In order to explore the novel circRNAs which participate in CRC progression, the present study analyzed a microarray dataset (GSE138589) in the Gene Expression Omnibus (GEO) database and identified a circRNA, circ_0006174, which is upregulated in CRC tissues. Some previous studies have reported that circ_0006174 promotes CRC progression and contributes to the chemoresistance of doxorubicin (8,9). Nevertheless, its role and mechanism in CRC remains to be elucidated.

miRNAs are ncRNA transcripts with a length of ~22 nucleotides, which can be used as promising downstream targets of several ncRNAs such as lncRNAs and circRNAs (10). miRNAs participate in tumorigenesis by binding to the 3′ UTR of target genes and repressing expression (10). The bioinformatics analysis of the present study suggested that miR-1205 had the potential complementary binding sites with circ_0006174. miR-1205 is reported to take part in the progression of diverse cancers, including CRC. For instance, miR-1205 modulates the cell cycle and impedes cell growth of CRC cells by targeting TRIM44 (11). miR-1205 can target CRK such as proto-oncogene (CRKL) to repress CRC progression (12). calcium-binding epidermal growth factor domain-containing protein 1 (CCBE1) is vital in the development of lymphatic vessels. CCBE1 is reported to enhance CRC metastasis by modulating the TGF-β signaling pathway (13). The bioinformatics analysis of the present study suggested a potential binding site between miR-1205 and CCBE1 3′UTR. Nonetheless, the miR-1205/CCBE1 axis in CRC is undefined.

The present study identified a circRNA, circ_0006174, which is substantially upregulated in CRC tissues and cell lines. Circ_0006174 was found to adsorb miR-1205 to up-modulate CCBE1 expression and facilitate CRC progression. This implies that circ_0006174 is a potential diagnostic biomarker and therapy target for CRC treatment.

## Materials and methods

### Participants and tissue cases

Between May 2018 and May 2020, CRC tissues and para-cancerous tissues (3 cm from the margin of tumor tissues) of 68 Chinese CRC patients (males:females, 31:37; median age at diagnosis, 60.8 years; age range, 45–72 years) were available from Guizhou Provincial People's Hospital and all specimens were determined by pathologists. None of the patients had received chemotherapy or radiotherapy or other anti-cancer treatments before surgery. The tissue samples were frozen in liquid nitrogen immediately after removal. This research was authorized by the Ethics Committee of Guizhou Provincial People's Hospital (approval no. 201802004) and complied with the Declaration of Helsinki. Prior to surgery, each subject completed a written informed consent form.

### Cell culture

Human CRC cell lines (HCT116, SW620, SW480 and HT29) and the immortalized colonic epithelial cell line NM460 were procured from ATCC. All cells were cultured in DMEM (Gibco; Thermo Fisher Scientific, Inc.) containing 10% FBS (Gibco; Thermo Fisher Scientific, Inc.), 100 U/ml penicillin and 100 µg/ml of streptomycin at 37°C with 5% CO_2_.

### Oligonucleotide transfection

Guangzhou RiboBio Co., Ltd. synthesized two small interfering RNA (siRNA) oligonucleotides targeting human circ_0006174 [si-circ_0006174#1 (50 nM): 5′-GACAGGCAAAATCCTCAATGA-3′ and si-circ_0006174#2 (50 nM): 5′-GCAACTGACAGGCAAAATCCT-3′] and siRNA negative control (50 nM) (5′-CCATTTACCCGAACGGCAA-3′), circ_0006174 short hairpin RNA [sh-circ_0006174 (80 nM): 5′-GCATCCATCACTCCAGCATCA-3′] and corresponding control [sh-NC (80 nM): 5′-TTCTCCGAACGTGTCACGT-3′], miR-1205 mimics (50 nM) (5′-UCUGCAGGGUUUGCUUUGAG-3′) and miR-1205 inhibitors (50 nM) (5′-CUCAAAGCAAACCCUGCAGA-3′) and miR-NC (50 nM) (5′-UCACAACCUCCUAGAAAGAGUAGA-3′) and inhibitor NC (50 nM) (5′-CAGUACUUUUGUGUAGUACAAA-3′). When the cultured cells reached 80% confluence, the CRC cells were planted into a 6-well plate and the above oligonucleotides were transfected into the cell line using Lipofectamine^®^ 2000 (Invitrogen; Thermo Fisher Scientific, Inc.). Transfection was performed at room temperature for 6 h. The cells were cultured for 24 h before subsequent experiments.

### Reverse transcription-quantitative (RT-q) PCR

Cells (5×10^4^/well) were cultured in 6-well plates. According to the manufacturers' protocols, total RNA was separated from tissues and cell lines using the TRIzol reagent (Thermo Fisher Scientific, Inc.) and a PrimeScript RT reagent kit (Takara Biotechnology Co., Ltd.) was utilized for reverse transcription to synthesize cDNA. Finally, a SYBR Green Premix Ex Taq kit (Takara Biotechnology Co., Ltd.) was utilized to conduct RT-qPCR and RNA relative expression was analyzed using the 2^−ΔΔCq^ technique (14) and GAPDH and U6 were employed as internal controls. The PCR reaction was as follows: 95°C for 5 min, followed by 40 cycles at 95°C for 30 sec, 60°C for 30 sec and elongation at 72°C for 15 sec. The following are the primer sequences: circ_0006174, 5′-GCAACTGACAGGCAAAATCC-3′ (forward) and 5′-AGTTGTGGTGGTGGAGGAAG-3′ (reverse); miR-1205, 5′-CTGCAGGGTTTGCTTTGAGG-3′ (forward) and 5′-CTCCAGAACAGGGTTGACAGG-3′ (reverse); CCBE1, 5′-TACCGATATGACCGGGAGAG-3′ (forward) and 5′-AGCTGCCCAAGGTATTGATG-3′ (reverse); U6, 5′-GACTATCATATGCTTACCGT-3′ (forward) and 5′-GGGCAGGAAGAGGGCCTAT-3′ (reverse); GAPDH, 5′-CTTTGGTATCGTGGAAGGACTC-3′ (forward) and 5′-GTAGAGGCAGGGATGATGTTCT-3′ (reverse).

### Subcellular fractionation

A PARIS kit (Invitrogen; Thermo Fisher Scientific, Inc.) was adopted for subcellular fractionation with U6 as a nuclear control and GAPDH as a cytoplasmic control. RT-qPCR was then conducted to examine relative expression in the subcellular fractionation respectively.

### CCK-8 method

Cells (2×10^3^) were inoculated into each well of a 96-well plate and 10 µl of CCK-8 reagent (Dojindo Laboratories, Inc.) was added to each well on day 1, 2, 3 and 4 and then the cells were incubated at 37°C for 1 h. Subsequently, a microplate reader (Bio-Rad Laboratories, Inc.) was employed to analyze the absorbance at 450 nm wavelength of each well.

### TUNEL assay

Cell apoptosis assay was performed using the TUNEL Apoptosis Assay kit (Beyotime Institute of Biotechnology). Cells were cultured in 24-well plates (1×10^5^ cells per well) and fixed with 4% paraformaldehyde for 15 min at 25°C. TUNEL solution was added to each well at 37°C for 60 min. After rinsing with phosphate buffer saline (PBS), the cells were stained with DAPI for 5 min at 37°C. The slides were mounted using Fluoromount-G^™^ (Thermo Fisher Scientific, Inc.). Three random fields of view were captured with a fluorescence microscope (Olympus Corporation) and the percentage of TUNEL-positive cells was calculated.

### Transwell assay

Transwell inserts (8-µm pore size; Corning, Inc.) were used for cell migration and invasion assays. For cell migration assay 5×10^4^ CRC cells in serum-free medium were planted in the top compartment of the Transwell compartment. Subsequently, 600 µl of medium containing 10% FBS was added to the lower compartment. Following incubation for 24 h at 37°C, the cells in the top compartment were removed. The cells on the lower surface of the filter were fixed with 95% ethanol and stained with 0.1% crystal violet for 15 min at room temperature. Then a light microscope (Olympus Corporation) was used for counting the cells. The bottom of the filter was pre-coated with Matrigel (1:10; BD Biosciences) for cell invasion experiments. The Matrigel-coated plate was incubated for 30 min at 37°C to polymerize the Matrigel. The remaining processes were identical to those used in the cell migration assay.

### Dual-luciferase reporter gene assay

Wild-type and mutant-type sequences of circ_0006174 or CCBE1 3′ UTR containing miR-1205 putative complementary binding sequences were designed by Promega Corporation, which were subsequently cloned into psiCHECKTM-2-luciferase reporter plasmid (Promega Corporation) and co-transfected with miR-1205 mimics (5′-UCUGCAGGGUUUGCUUUGAG-3′) (Guangzhou RiboBio Co., Ltd.) or miR-NC (5′-UCACAACCUCCUAGAAAGAGUAGA-3′) (Guangzhou RiboBio Co., Ltd.) into CRC cells using Lipofectamine^®^ 2000 (Invitrogen; Thermo Fisher Scientific, Inc.) for 24 h. The relative luciferase activity of the cells in each group was measured 48 h later with a dual-luciferase assay system (Promega Corporation). *Renilla* luciferase activity was used to normalize the firefly luciferase activity.

### Western blotting

RIPA lysis buffer (Beyotime Institute of Biotechnology) was utilized to isolate the total protein from the cells and the concentration of protein was analyzed using the BCA detection kit (Beyotime Institute of Biotechnology). Sodium dodecyl sulfate polyacrylamide gel electrophoresis using a 10% gel was utilized to separate 30 µg protein sample before the protein samples were transferred to PVDF membranes (MilliporeSigma). The PVDF membrane was blocked with 5% skimmed milk for 1.5 h at room temperature and then incubated with anti-CCBE1 (1:1,000; HPA041361; MilliporeSigma), anti-Ki67 (1:1,000; cat. no. ab16667; Abcam), anti-c-Myc (1:1,000; cat. no. ab32072; Abcam), anti-cyclin D1 (1:1,000; cat. no. ab134175; Abcam) and anti-GAPDH (1:1,000; cat. no. ab9485; Abcam) overnight at 4°C and then the membrane was incubated at room temperature for 1 h with the horseradish peroxidase-conjugated secondary antibody (1:2,000; cat. no. ab150077; Abcam). To identify protein bands, the ECL luminescence reagent (Thermo Fisher Scientific, Inc.) was utilized. Semi-quantitative analysis was conducted using ImageJ software (version 1.8.0; National Institutes of Health).

### Tumor xenografts and lung metastasis in mice

Animal experiments were authorized by the Animal Care Committee of Guizhou Provincial People's Hospital (approval no. 202109010) and conducted in accordance with the guidelines of National Health Institute. A total of 10 four-week-old female BALB/c nude mice (weight, 18–20 g; Guizhou Yikeda Biotechnology Co. Ltd.) were maintained under standard laboratory conditions at 25°C with 12-h light/dark cycles, 60% humidity and free access to food and water. The mice werte randomly divided into two groups. SW620 cells were transfected with sh-NC or sh-circ_0006174 and resuspended in PBS. Then, the cells were injected into the tail vein of each nude mice (1×10^6^ cells per mouse). After 30 days, the mice were decapitated and the lungs were removed and fixed in 4% paraformaldehyde at 4°C for 24 h and embedded in paraffin. Hematoxylin and eosin (H&E) staining was performed to evaluate the formation of metastatic nodules. Sections (5-µm thick) were deparaffinized in xylene, rehydrated in graded alcohol solution and stained with hematoxylin solution for 30 min, and then washed with water and counterstained with eosin for 5 min at 25°C. Images were captured using a microscope (Olympus Corporation).

### Bioinformatics

The eligible circRNAs data set (GSE138589) (15) was obtained from the GEO and included data from 6 CRC tissues and 6 normal tissues. The raw microarray data were processed using GEO2R. The Circular RNA Interactome database (https://circinteractome.nia.nih.gov/index.html) was used to explore the downstream miRNAs of circ_0006174 and the target genes of miR-1205. Gene Expression Profiling Interactive Analysis (GEPIA; http://gepia.cancer-pku.cn/) was used to analyze the relationship between CCBE1 expression and overall survival. The LinkedOmics (http://www.linkedomics.org/) online platform (16) was used to analyze the signal pathways associated with CCBE1 in CRC.

### Statistical analysis

All the experiments were performed in triplicate and the data were shown as mean ± standard deviation. Statistical analysis was executed using SPSS 17.0 data software (SPSS Inc.) and GraphPad Prism V7.0 (GraphPad Software, Inc.) was adopted for plotting. Paired (for matched data, such as tumor vs. normal tissue) and unpaired (all other comparisons between two groups) Student's t-test was used, while one-way ANOVA was performed to examine difference among three or more groups, with Turkey's post-hoc test. Correlation analysis was conducted using Pearson's correlation analysis. χ^2^ test was performed to analyze the association between patients' pathological characteristics and the expression level of circ_0006174 or CCBE1.

## Results

### Circ_0006174 is markedly upregulated in CRC tissues and cell lines

**Figure 1.**
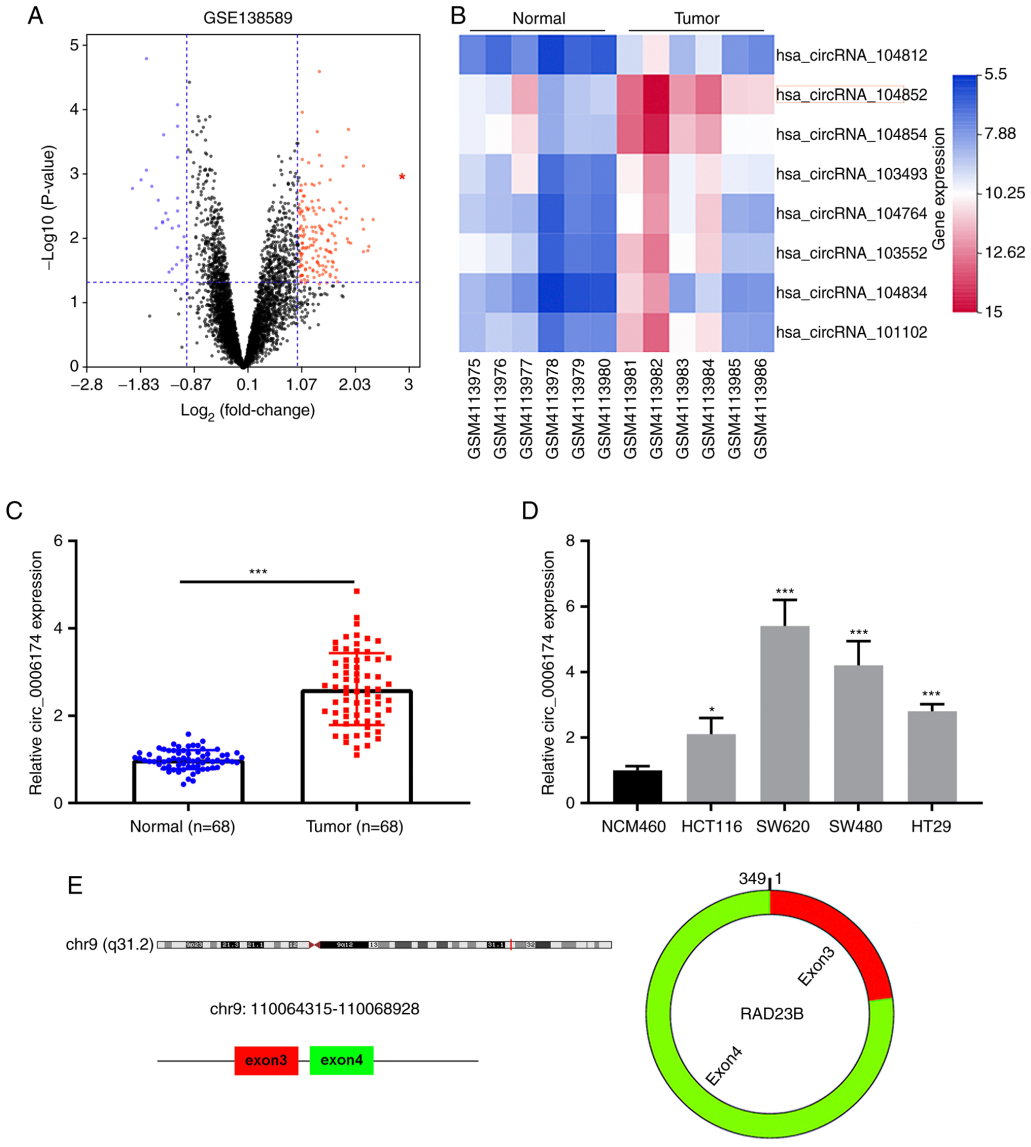
circ_0006174 is upregulated in CRC tissues and cell lines. (A) Volcano map was used to evaluate the circRNAs that were markedly upregulated and downregulated in the CRC tissue in the GEO dataset (GSE138589). (B) Heat map was adopted to show the top eight circRNAs that were markedly upregulated in CRC. (C) RT-qPCR was used to detect circ_0006174 expression in 68 pairs of CRC tissues and paracancerous non-tumor tissues. (D) RT-qPCR was used to analyze circ_0006174 expression in normal human colonic epithelial cell line and CRC cell lines (HCT116, SW620, SW480 and HT29). (E) Diagram of the circular structure of circ_0006174. *P<0.05; ***P<0.001. CRC, colorectal cancer; circRNAs, circular RNA; GEO, Gene Expression Omnibus; RT-qPCR, reverse transcription-quantitative PCR.

The GEO database circRNA microarray GSE138589 was used to analyze the differential circRNA expression in CRC tissues and adjacent tissues using the cut-of criteria (|log_2_(fold change)|>1 and P<0.05; Fig. 1A). Fig. 1B displayed the top eight circRNAs that were markedly upregulated in CRC tissues. Then circ_104852 (circ_0006174) was selected for followed-up analysis. RT-qPCR was then performed to analyze circ_0006174 expression in 68 pairs of CRC tissues and paracancerous tissues. The data showed that circ_0006174 expression in CRC tissues was markedly higher than that in adjacent tissues (Fig. 1C). Additionally, compared with immortalized human colonic epithelial cell lines, circ_0006174 expression was notably higher in CRC cell lines (HCT116, SW620, SW480 and HT29; Fig. 1D). As shown in Fig. 1E, circ_0006174 was a ring structure composed of the third and fourth exons of RAD23 Homolog B (RAD23B) gene.

Using the median circ_0006174 expression in CRC tissues, 68 CRC patients were divided into the overexpression group and the under-expression group (high expression group: n=34; low expression group: n=34) to analyze the relationship between circ_0006174 expression and the clinicopathological characteristics of CRC patients. It was observed that circ_0006174 overexpression was associated with larger tumor diameter (P=0.027) and higher T stage (P=0.014; Table I). The above data suggested that circ_0006174 was markedly upregulated in CRC tissues and perhaps implicated in CRC progression as an oncogene. Given that circ_0006174 had the highest expression in SW620 and SW480, these two cell lines were chosen for subsequent experiments.

### Knockdown of circ_0006174 markedly impedes the growth, migration and invasion of CRC cells

**Figure 2.**
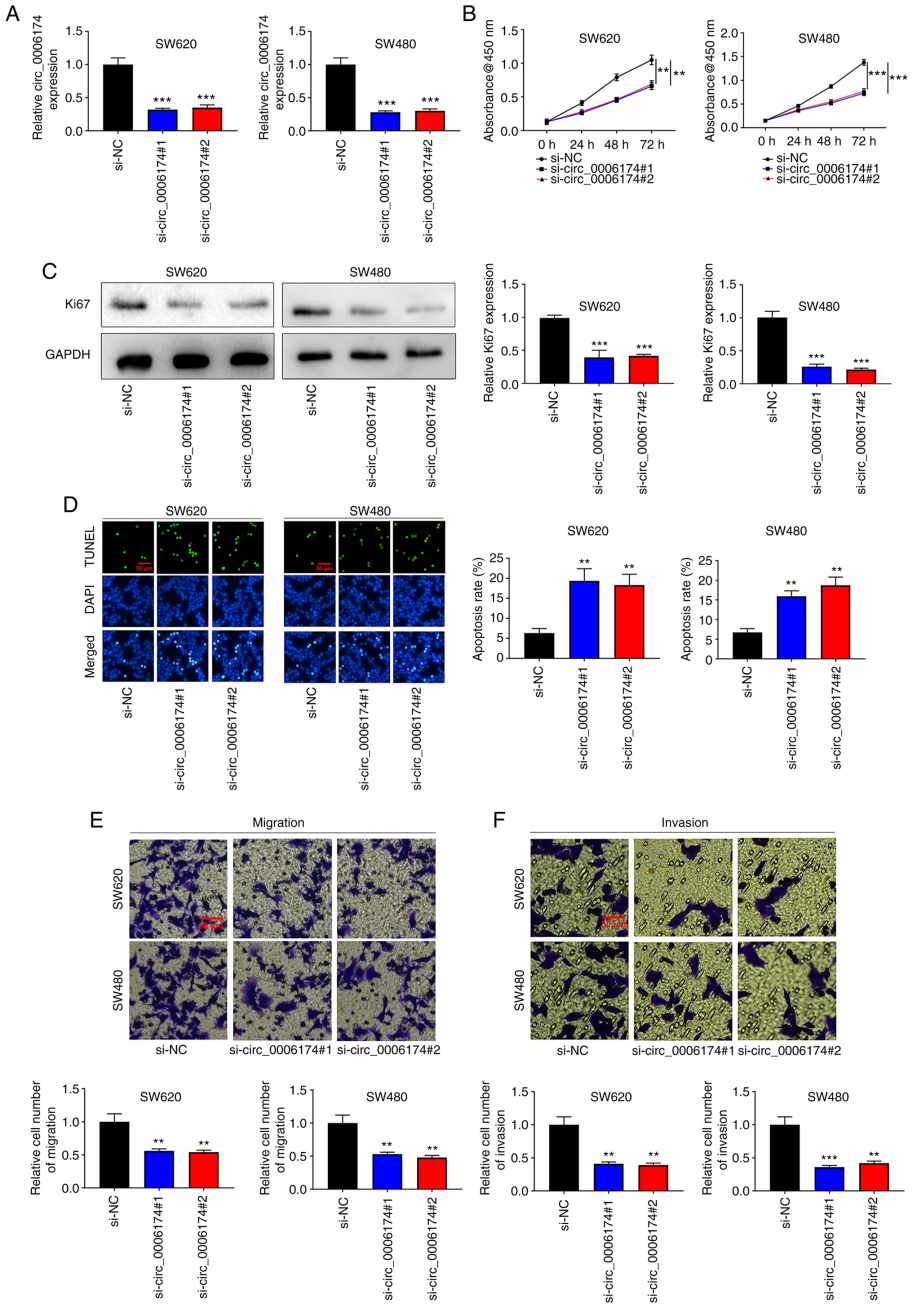
Knockdown of circ_0006174 significantly restrains the growth, migration and invasion of CRC cells. (A) RT-qPCR was performed to examine circ_0006174 expression after CRC cells were transfected with circ_0006174 siRNA. (B) CCK-8 test was implemented to examine cell growth after transfection with circ_0006174 siRNA. (C) Western blotting was used to detect Ki67 expression in CRC cells transfected with circ_0006174 siRNA. (D) TUNEL assay was used to examine cell apoptosis rate of CRC cells transfected with circ_0006174 siRNA. Transwell assay was exploited to examine cell (E) migration and (F) invasion of CRC cells transfected with circ_0006174 siRNA. **P<0.01 and ***P<0.001. circ, circular RNA; RT-qPCR, reverse transcription-quantitative PCR; CRC, colorectal cancer; si, small interfering; NC, negative control.

To understand the regulatory effects of circ_0006174 on the biological behavior of CRC cells, two circ_0006174 siRNAs (si-circ_0006174#1 and si-circ_0006174#2) were transfected into SW620 and SW480 cell lines and a circ_0006174 low expression model successfully constructed (Fig. 2A). The CCK-8 method was utilized to appraise cell growth. The data revealed that cell growth in the knocked down circ_0006174 group was markedly lower compared with the control group (Fig. 2B). Similarly, Ki-67, the proliferation-related protein, was significantly decreased in the knocked down circ_0006174 group (Fig. 2C). Additionally, the results of TUNEL assay revealed that the apoptosis rate of SW620 and SW480 cells was markedly promoted in the si-circ_0006174#1 and si-circ_0006174#2 group compared with the si-NC group (Fig. 2D). Transwell assay was employed to evaluate cell migration and invasion and the data showed that as opposed to the control group, the cell migration and invasion of si-circ_0006174#1 and si-circ_0006174#2 groups were markedly repressed (Fig. 2E and F). The above results suggested that circ_0006174 mediated the malignant phenotype of CRC cells.

### Circ_0006174 is the molecular sponge of miR-1205

**Figure 3.**
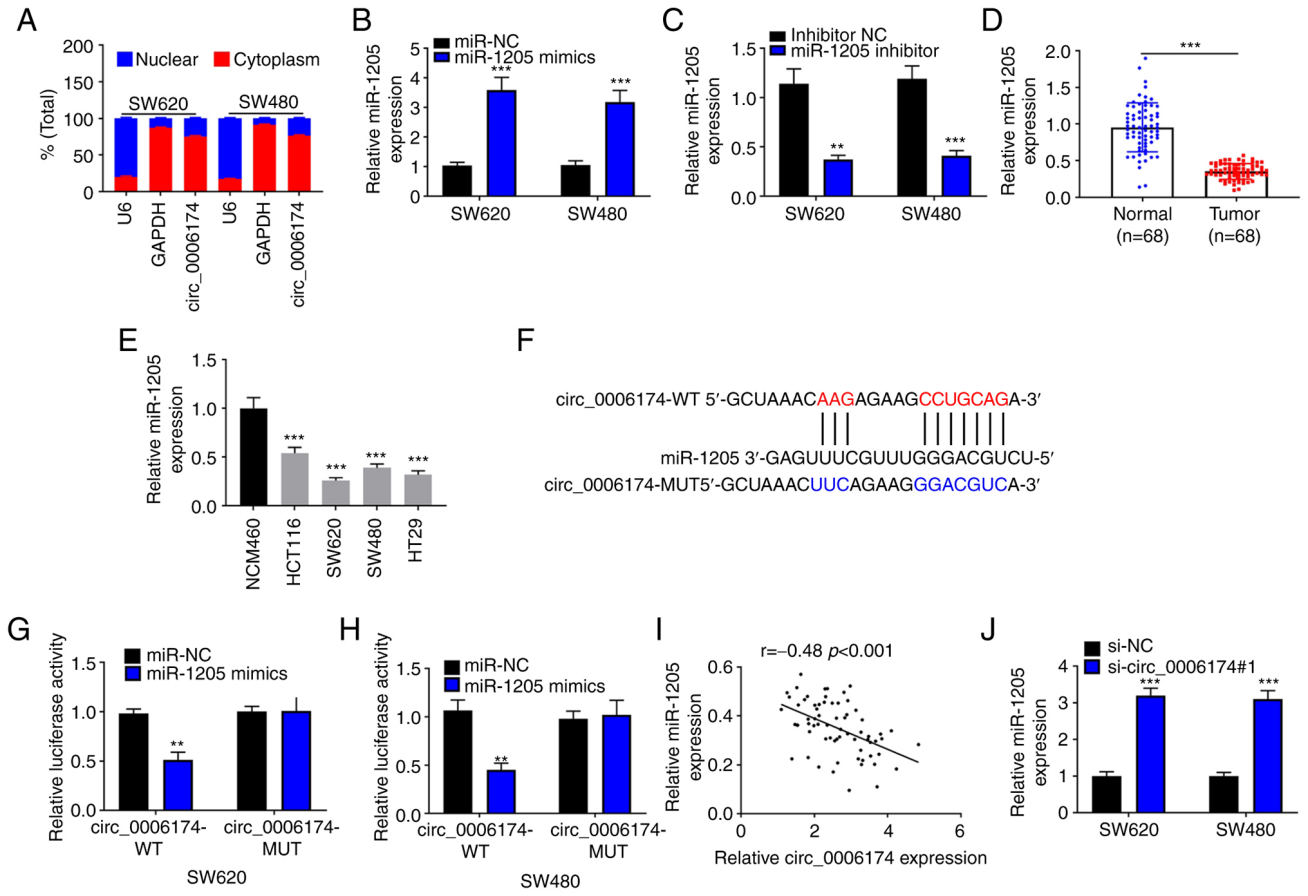
Circ_0006174 is the molecular sponge of miR-1205. (A) RT-qPCR was conducted to determine circ_0006174 expression in the cytoplasm and nucleus of CRC cells. RT-qPCR was applied to determine miR-1205 expression in SW620 and SW480 cells transfected with (B) miR-1205 mimics or (C) miR-1205 inhibitor. (D) RT-qPCR was applied to determine miR-1205 expression in 68 cases of CRC tissues and adjacent noncancerous tissue. (E) RT-qPCR was performed to detect miR-1205 expression in normal human colonic epithelial cell line and CRC cell lines (HCT116, SW620, SW480 and HT29). (F) The putative binding site of circ_0006174 for miR-1205 (circ_00061747-WT) and the designed mutant sequence (circ_0006174-MUT) were shown. (G and H) The dual-luciferase reporter gene experiment was performed to validate the direct binding between circ_0006174 and miR-1205. (I) Pearson's correlation coefficient was utilized to analyze the relationship between miR-1205 expression and circ_0006174 expression in CRC tissue. (J) RT-qPCR was performed to examine miR-1205 expression in CRC cell line transfected with circ_0006174 siRNA. **P<0.01 and ***P<0.001. circ, circular RNA; miR, microRNA; RT-qPCR, reverse transcription-quantitative PCR; CRC, colorectal cancer; WT, wild-type; MUT, mutant; NC, negative control.

**Figure 4.**
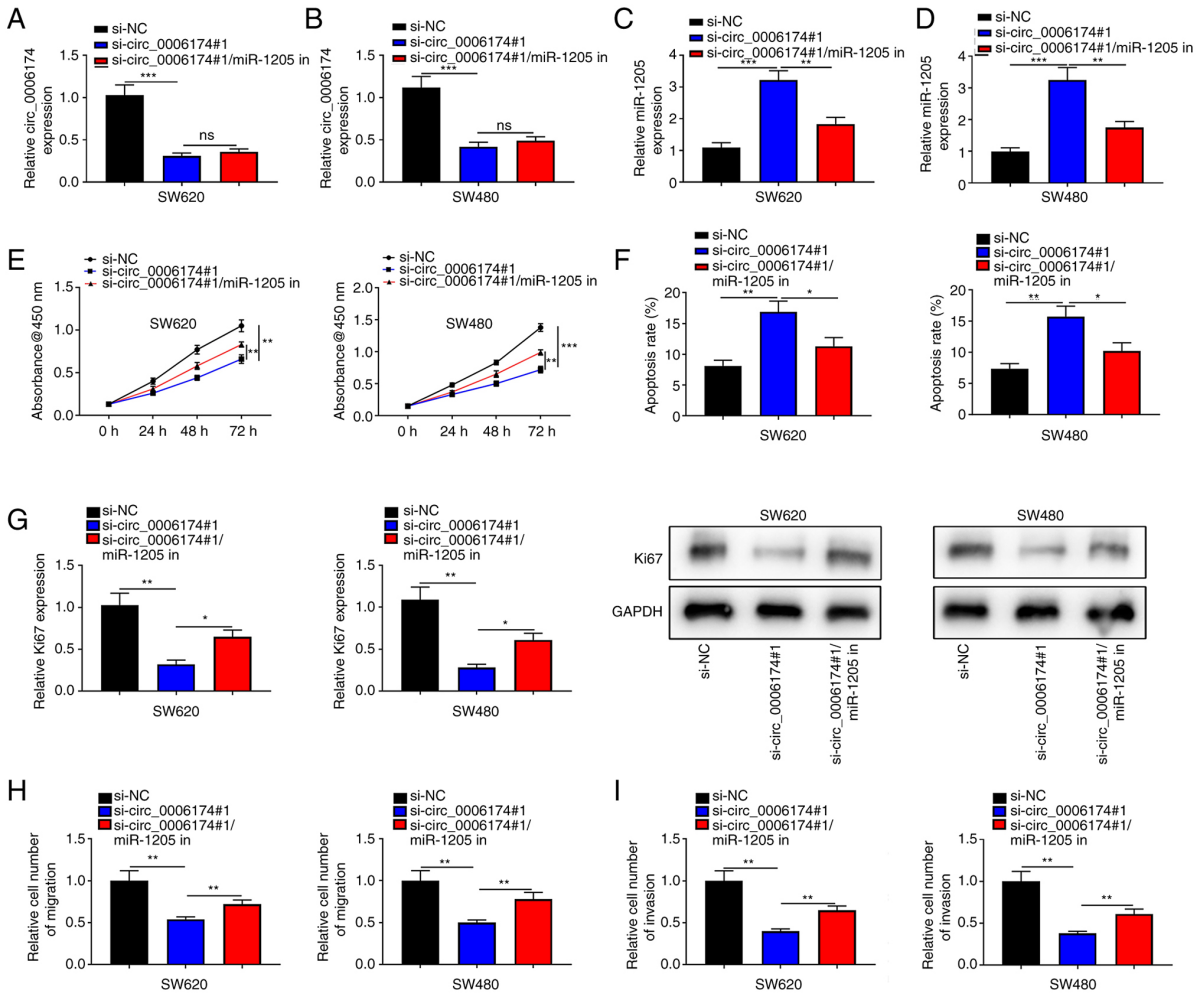
miR-1205 can reverse the effect of circ_0006174 on CRC cells. (A and B) RT-qPCR was used to detect the expression of circ_0006174 in CRC cells co-transfected with circ_0006174 siRNA and miR-1205 inhibitor. (C and D) RT-qPCR was used to detect the expression of miR-1205 in CRC cells co-transfected with circ_0006174 siRNA and miR-1205 inhibitor. (E) CCK-8 experiment was used to detect the growth of CRC cells co-transfected with circ_0006174 siRNA and miR-1205 inhibitor. (F) TUNEL assay was used to detect the apoptosis rate of CRC cells co-transfected with circ_0006174 siRNA and miR-1205 inhibitor. (G) Western blotting was used to detect the expression of Ki67 in CRC cells co-transfected with circ_0006174 siRNA and miR-1205 inhibitor. (H and I) Transwell method was performed to examine the (H) migration and (I) invasion of CRC cells co-transfected with circ_0006174 siRNA and miR-1205 inhibitor. *P<0.05, **P<0.01 and ***P<0.001. miR, microRNA; circ, circular RNA; CRC, colorectal cancer; RT-qPCR, reverse transcription-quantitative PCR; si, small interfering; NC, negative control; ns, not significant.

RT-qPCR suggested that circ_0006174 mainly existed in the cytoplasm of SW620 and SW480 (Fig. 3A). The transfection of miR-1205 mimics could significantly increase the expression of miR-1205 in SW620 and SW480 cells and the transfection of miR-1205 inhibitor decreased the expression of miR-1205 in SW620 and SW480 cells (Fig. 3B and C). Furthermore, RT-qPCR was performed to determine miR-1205 expression in CRC tissues and cell lines and the data showed that miR-1205 was under-expressed in CRC tissues and cell lines (Fig. 3D and E). Hence, circ_0006174 may be implicated in the modulation of downstream gene expression at the post-transcriptional level as ceRNAs. In the Circular RNA Interactome database, it was observed that miR-1205 contained a binding site complementary to circ_0006174 (Fig. 3F). To confirm the binding relationship between the two, a dual-luciferase reporter gene assay was conducted and the data confirmed that miR-1205 markedly repressed the luciferase activity of the cells in circ_0006174-wild-type (WT) group, while the luciferase activity of the cells in circ_0006174-mutant (MUT) group was unchanged (Fig. 3G and H). Pearson's correlation analysis showed that miR-1205 expression in CRC tissue was negatively correlate with circ_0006174 expression (Fig. 3I). miR-1205 expression was markedly promoted in si-circ_0006174#1 group (Fig. 3J). To validate that circ_0006174 functioned in CRC by adsorbing miR-1205, ‘rescue’ experiments were performed and the data showed that the co-transfection of miR-1205 inhibitors partly abolished the effects of circ_0006174 depletion on CRC cell growth, apoptosis, migration and invasion (Fig. 4). The above data implied that circ_0006174 was the molecular sponge of miR-1205 and circ_0006174 was oncogenic in CRC through regulating the expression of miR-1205.

### Circ_0006174 decoys miR-1205 to modulate CCBE1 expression

**Figure 5.**
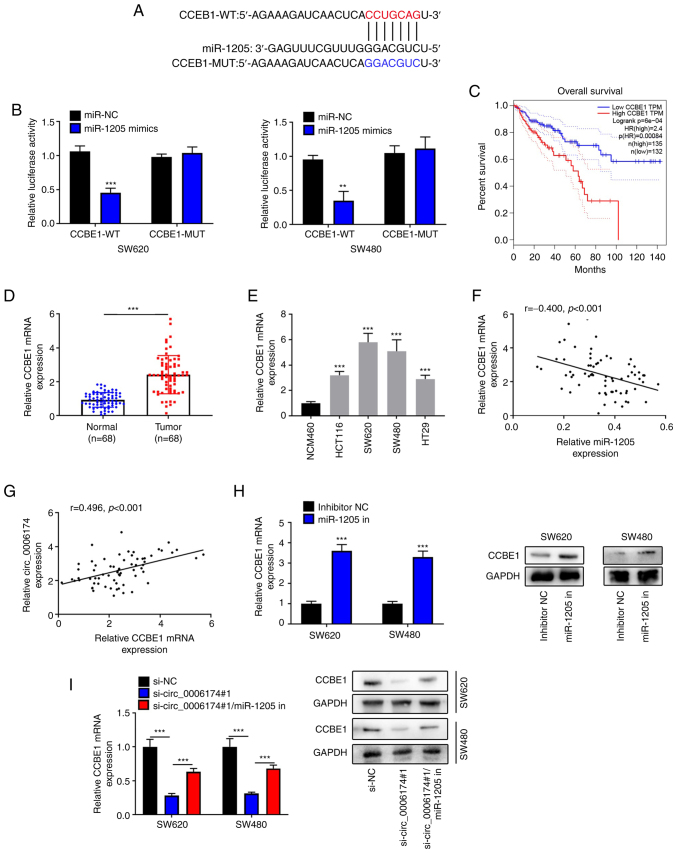
Circ_0006174 targets miR-1205 to modulate CCBE1 expression. (A) Bioinformatics was used to predict the binding site of CCBE1 3′UTR to miR-1205. (B) Dual-luciferase reporter gene experiment was used to confirm the binding sites between miR-1205 and CCBE1 3′UTR. (C) GEPIA database analyzed the relationship between CCBE1 expression and the survival rate of CRC patients. (D) RT-qPCR was utilized to examine CCBE1 mRNA5 expression in 68 cases of CRC tissues. (E) RT-qPCR was performed to determine CCBE1 mRNA expression in normal human colonic epithelial cell line and CRC cell lines (HCT116, SW620, SW480 and HT29). Pearson's correlation coefficient was used to analyze the association between (F) miR-1205 expression and CCBE1 expression and (G) CCBE1 expression and circ_0006174 expression in CRC tissue. (H) RT-qPCR and western blotting experiments were employed to determine CCBE1 mRNA and protein expression in CRC cells following transfection with miR-1205 inhibitor. (I) RT-qPCR and western blotting were performed to determine CCBE1 mRNA and protein expression after the cells were co-transfected with circ_0006174 siRNA and miR-1205 inhibitor. **P<0.01 and ***P<0.001. circ, circular RNA; miR, microRNA; CCBE1, calcium-binding epidermal growth factor domain-containing protein 1; GEPIA, Gene Expression Profiling Interactive Analysis; CRC, colorectal cancer; RT-qPCR, reverse transcription-quantitative PCR; WT, wild-type; MUT, mutant; NC, negative control.

Next, StarBase database was utilized to detect the downstream target of miR-1205 and it was revealed that CCBE1 was the most potential target gene (Fig. 5A). Dual-luciferase reporter gene experiments confirmed that the luciferase activity of CRC cells co-transfected with the luciferase plasmids psiCHECKTM-2-CCBE1-WT and miR-1205 mimics was markedly reduced compared with the control group, while no obvious change in the luciferase activity of the cells was observed in the cells co-transfected with CCBE1-MUT and miR-1205 mimics (Fig. 5B). This indicated that miR-1205 could bind with CCBE1 3′UTR. GEPIA database suggested that the survival rate of CRC patients with high CCBE1 expression was worse (Fig. 5C). The data of RT-qPCR showed that CCBE1 was markedly upregulated in CRC tissues and cell lines (Fig. 5D and E). Based on the expression of CCBE1 in CRC tissues, the 66 CRC patients were divided into high expression group and low expression group. It was revealed that CCBE1 expression was not associated with clinicopathological features of the patients (Table II). Notably, circ_0006174 expression and miR-1205 expression in CRC tissues were negatively correlated and there was a positive correlation between circ_0006174 expression and CCBE1 expression (Fig. 5F and G). The findings of RT-qPCR and western blotting suggested that inhibition of miR-1205 markedly elevated CCBE1 expression in CRC cell lines (Fig. 5H) and the transfection of miR-1205 inhibitor counteracted the suppressive effect of knocking down circ_0006174 on CCBE1 expression (Fig. 5I). The above data confirmed that in CRC cell lines, circ_0006174 positively modulated CCBE1 expression by targeting miR-1205.

### Circ_0006174 upregulates Wnt pathway-related proteins and promotes lung metastasis in vivo

**Figure 6.**
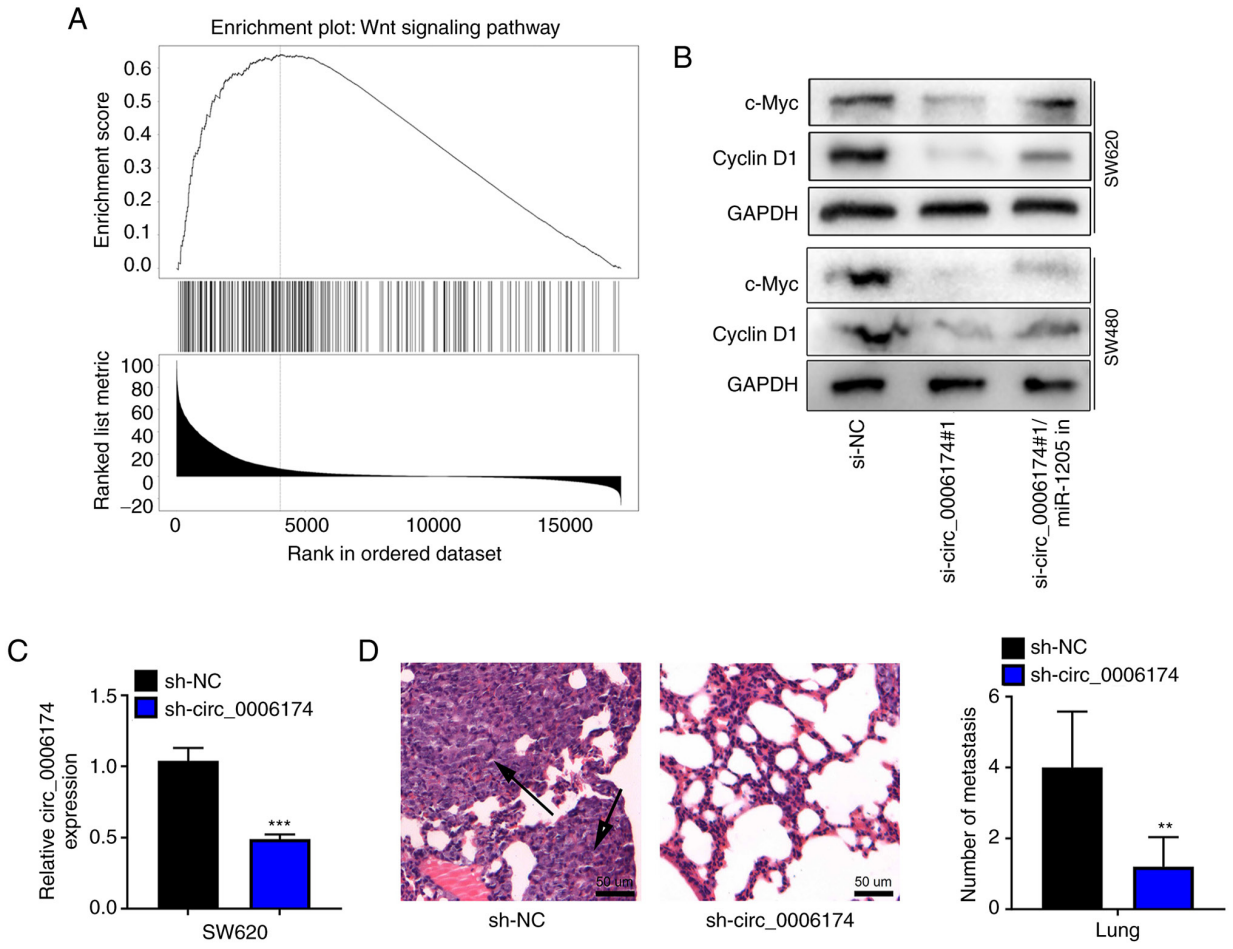
circ_0006174 upregulates Wnt signal pathway-related proteins in CRC cells and promotes lung metastasis in vivo. (A) Gene set enrichment analysis suggested that CCBE1 was positively related to Wnt signal pathway in CRC. (B) Western blotting was used to detect Wnt signal pathway-related proteins (c-Myc and cyclin D1) in CRC cells transfected with circ_0006174 siRNA and miR-1205 inhibitor. (C) RT-qPCR was used to detect circ_0006174 expression in sh-circ_0006174 group. (D) The number of lung metastases were calculated in nude mice. The metastatic nodules were indicated by the arrows. **P<0.01 and ***P<0.001 vs. sh-NC. circ, circular RNA; CRC, colorectal cancer; CCBE1, calcium-binding epidermal growth factor domain-containing protein 1; circ, circular RNAs.

To further identify the downstream mechanism of CCBE1 in CRC, gene set enrichment analysis was performed with LinkedOmics database and the results showed that CCBE1 was positively associated with the activation of Wnt pathway (Fig. 6A). Western blot assay confirmed that the expression levels of c-Myc and cyclin D1 were significantly downregulated in the si-circ_0006174#1 group compared with the si-NC group and the transfection of miR-1205 inhibitor reversed the inhibitory effect of circ_0006174 knockdown on the expression of c-Myc and cyclin D1 in SW620 and SW480 cells (Fig. 6B). RT-qPCR suggested that the expression of circ_0006174 in sh-circ_0006174 group was decreased compared with the sh-NC group (Fig. 6C). H&E staining further showed that the number of lung metastatic nodules were fewer and the metastatic nodules were much smaller in sh-circ_0006174#1 group (Fig. 6D). These results further supported that circ_0006174 was an oncogenic circRNA in CRC.

## Discussion

Reportedly, circRNAs are closely associated with CRC carcinogenesis and development (17). circ_0006174 is a circRNA which is transcribed from chromosome chr9:110064315-110068928+. A previous study reports that circ_0006174 is highly expressed in CRC tissues and cells that and circ_0006174 can promotes CRC progression via sponging miR-138-5p and upregulating MACC1 (8). Another study reports that the exosomal circ_0006174 promotes doxorubicin resistance of CRC cells (9). In the present study, by analyzing GEO microarray data, it was found that circ_0006174 was significantly upregulated in CRC tissues. This result was also confirmed in the 68 specimens of CRC patients. Moreover, circ_0006174 expression in CRC cell lines was notably higher than that in normal human colonic epithelial cell lines. Additionally, circ_0006174 overexpression was associated with larger tumor diameter and higher clinical stage of CRC patients. Furthermore, the loss-of-function experiments results implied that circ_0006174 could enhance cells proliferation, metastasis and inhibit cell apoptosis rate *in vitro* and promote lung metastasis *in vivo*. These findings revealed that circ_0006174 served as an oncogenic factor to participant in CRC progression.

The ceRNA hypothesis provides a new mechanism of RNA interaction. circRNA can function as ceRNA to modulate gene expression by targeting miRNA (18). The present study revealed that circ_0006174 was mainly distributed in the cytoplasm, so it is hypothesized that circ_0006174 serves as a ceRNA. Bioinformatics and dual-luciferase reporter gene assay showed that miR-1205 could directly and specifically bind with circ_0006174. As a tumor suppressor, miR-1205 restrains the development of diverse malignancies. For instance, miR-1205 represses NSCLC progression by downregulating KRAS expression (19). In gliomas, miR-1205 targets BATF3 to restrain cell growth and invasion (20). In ovarian cancer, the miR-1205/SH2D3A axis modulates cell growth, migration and invasion (21). Reportedly, miR-1205 is significantly downregulated in CRC tissues and it represses the growth and metastasis of CRC cells by targeting MYO6 (22). The findings of the present study showed that miR-1205 was markedly downregulated in CRC tissues and cell lines and was negatively correlated with circ_0006174 expression; additionally, circ_0006174 knockdown in CRC cell lines markedly enhanced miR-1205 expression; besides, circ_0006174 expression and miR-1205 expression in CRC tissues were negatively correlated; miR-1205 inhibitor counteracted the suppressing effect of knocking down circ_0006174 on the growth, migration and invasion of CRC cells. Hence, it was validated that circ_0006174 was the molecular sponge of miR-1205 and circ_0006174 works in CRC cells by modulating miR-1205.

Reportedly, CCBE1 can not only modulate extracellular matrix remodeling, but also are crucial in angiogenesis and lymphangiogenesis (23). Accumulating research has demonstrated that CCBE1 is vital in cancer biology. CCBE1 is identified as a target gene of miR-330-3p, which is related to the aggressive phenotype of breast cancer (24). In gastrointestinal stromal tumor patients, the patients with CCBE1 overexpression have worse overall survival and relapse-free survival compared with patients with CCBE1 under-expression (25). In CRC, CCBE1 overexpression is reported to be markedly associated with tumor differentiation, lymph node metastasis, vascular invasion, liver metastasis and TNM stage of CRC patients (26). Notably, the present study observed that there was no significant association between CCBE1 expression level and the characteristics of the patients and this is probably due to the heterogeneity of the tissue samples. CCBE1 is demonstrated to facilitate lymphangiogenesis and lymphatic metastasis in CRC (13). In the present study, CCBE1 was identified as the target gene of miR-1205. Bioinformatics analysis revealed that CCBE1 was overexpressed in CRC tissues and was associated with the unfavorable prognosis of CRC patients. Furthermore, it was also revealed that CCBE1 was markedly upregulated in CRC tissues and cell lines and circ_0006174 upregulated CCBE1 expression by inhibiting miR-1205. The present study partly explains the mechanism of CCBE1 dysregulation in CRC.

As one of the most important intracellular signal pathways, Wnt/β-catenin signaling is associated with diverse cellular processes, such as cell proliferation, differentiation, migration, survival and metastasis. Dysregulated Wnt pathway has been reported to promote CRC progression. For instance, lncRNA SLCO4A1-AS1 promotes CRC cell proliferation, migration, invasion and epithelial-mesenchymal transition via the Wnt/β-catenin pathway (27). SNHG16 is regulated by the Wnt pathway and plays an oncogenic role in CRC (28). In the present study, it was confirmed that CCBE1 was associated with the activation of Wnt pathway in CRC. Knockdown of circ_0006174 could decrease Wnt-related proteins by regulating miR-1205 in CRC cells. However, how circ_0006174/miR-1205/CCBE1 axis modulates the activation of Wnt signaling requires further investigation.

The present study has some limitations. For example, whether CCBE1 promotes the proliferation and metastasis of CRC cells through Wnt pathway needs to be verified. The other potential target genes of miR-1205 in CRC remains largely unknown. which will be investigated in the future.

In summary, the present study elaborated on the biological role of circ_0006174 in CRC cells and revealed the role of the circ_0006174/miR-1205/CCBE1 axis in CRC progression. Hence, circ_0000326 is likely to be a novel target for CRC therapy.

## Figures and Tables

**Table I.** Correlations between hsa_circ_0006174 expression and clinical features in patients with colorectal cancer.

		circ_0006174		
			
Features	n	High (n=34)	Low (n=34)	P-value
Age, years				
≥60	36	19	17	0.808
<60	32	15	17	
Sex				
Male	31	18	13	0.796
Female	37	16	21	
Clinical stage				
T3-T4	37	24	13	0.014^a^
T1-T2	31	10	21	
Tumor size, cm				
≥3	38	24	14	0.027^a^
<3	30	10	20	

a P<0.05; circ, circular RNA.

**Table II.** Correlations between CCBE1 expression and clinical features in patients with colorectal cancer.

		CCBE1		
			
Features	n	High (n=34)	Low (n=34)	P-value
Age, years				
≥60	36	19	17	0.627
<60	32	15	17	
Sex				
Male	31	16	15	0.807
Female	37	18	19	
Clinical stage				
T3-T4	37	20	17	0.465
T1-T2	31	14	17	
Tumor size, cm				
≥3	38	18	20	0.625
<3	30	16	14	

CCBE1, calcium-binding epidermal growth factor domain-containing protein 1.

## Data Availability

The datasets used and/or analyzed during the current study are available from the corresponding author on reasonable request.
